# Psychiatric disorders with antiseizure medications in children: an analysis of the FDA adverse event reporting system database

**DOI:** 10.1186/s42494-025-00223-5

**Published:** 2025-05-23

**Authors:** Jianxiong Gui, Lingman Wang, Linxue Meng, Xiaofang Zhang, Jiannan Ma, Li Jiang

**Affiliations:** 1 Children’s Medical Center, Peking University First Hospital, Beijing, 100034 China; 2 Department of Neurology, National Clinical Research Center for Child Health and Disorders, Ministry of Education Key Laboratory of Child Development and Disorders, Chongqing Key Laboratory of Child Neurodevelopment and Cognitive Disorders, Children’s Hospital of Chongqing Medical University, Chongqing, 400014 China

**Keywords:** Antiseizure medications, Adverse events, Children, FAERS database, Psychiatric disorders

## Abstract

**Background:**

Epilepsy is a chronic neurological disorder marked by a persistent tendency to generate seizures, leading to substantial cognitive, behavioral, and psychosocial consequences. This study investigated psychiatric disorder-related adverse events (AEs) associated with antiseizure medications (ASMs) in children using the Food and Drug Administration Adverse Event Reporting System (FAERS) database.

**Methods:**

This study conducted a comprehensive analysis of FAERS data from 2004 to 2024, focusing on psychiatric AEs in children with epilepsy or seizures treated with ASMs. Signal values were computed using reporting odds ratio (ROR), proportional reporting ratio (PRR), Bayesian Confidence Propagation Neural Network (BCPNN), and Multi-item Gamma Poisson Shrinker (MGPS).

**Results:**

A total of 2539 preferred terms (PTs) were included, involving 25 system organ classifications (SOCs). Nervous system, skin and subcutaneous tissue, and psychiatric disorders are the three most common SOCs for ASMs in children. There were 24 ASMs, whose AEs involved psychiatric disorders, totaling 110 PTs and 214 drug-PT relationships. Psychotic symptoms (notably lorazepam and topiramate, *n* = 116 and 109), substance dependence and abuse (notably pregabalin and clonazepam, *n* = 291 and 110), and the other neuropsychiatric symptoms (notably levetiracetam and valproic acid, *n* = 70 and 62) were the common types of psychiatric disorder-related AEs of ASMs in children. A total of nine ASMs (brivaracetam, clonazepam, diazepam, eslicarbazepine, gabapentin, lamotrigine, lorazepam, perampanel, and tiagabine) were associated with suicidal and self-injurious behavior in children.

**Conclusions:**

This study highlights psychiatric AEs of ASMs in children, offering critical insights to improve clinical medication practices and enhance treatment safety. Further research with broader clinical data is needed to promote safe and rational medication use.

**Supplementary Information:**

The online version contains supplementary material available at 10.1186/s42494-025-00223-5.

## Background

Epilepsy is a chronic neurological disorder characterized by a persistent predisposition to generate epileptic seizures and the resulting neurobiological, cognitive, psychological, and social implications of this condition [1]. Epilepsy affects more than 70 million individuals worldwide, with nearly 80% residing in low- and middle-income nations [2]. In most low-income countries, over 75% of individuals experiencing active epilepsy do not have access to adequate treatment [3]. The global burden of epilepsy report estimates that annually, epilepsy accounts for 13 million disability-adjusted life years (DALYs), making it a significant global health concern [4].

Epilepsy has a high incidence in early childhood [5]. At the population level, the incidence exhibits a bimodal distribution, with the highest age-specific rate of 460 cases per 100,000 individuals observed among children under 5 years old and adults over 65 years old [6]. The incomplete development of the brain may result in seizures having even more profound implications for children's cognitive and behavioral development [7].

The cornerstone of treatment for most patients with epilepsy lies in the administration of antiseizure medications (ASMs). Approximately 70% of individuals who have recently been diagnosed with epilepsy can effectively manage their seizures with a single medication [2]. Despite the wide range of available drugs globally, the selection of ASMs should consider the specific type of epilepsy and its corresponding syndrome, as well as the patient's age, gender, comorbidities, and potential drug interactions [8]. Currently, ASMs are primarily categorized into three generations: the first generation, the second generation, and the third generation. The first-generation ASMs, such as phenytoin, carbamazepine, and valproic acid, have been extensively utilized in clinical practice over the past few decades; however, they exhibit significant side effects including cognitive dysfunction and behavioral issues [9–11]. The second-generation ASMs like lamotrigine, topiramate, and levetiracetam have demonstrated improvements in terms of efficacy and safety; nevertheless, certain adverse effects persist such as skin rashes, weight fluctuations, and mood swings [12, 13, 14]. The third-generation ASMs such as lacosamide, eslicarbazepine, and pregabalin have exhibited favorable outcomes in treating specific types of epilepsy; nonetheless, their long-term safety necessitates further investigation [15, 16]. According to a global survey conducted by the International League Against Epilepsy (ILAE) in 2022, it was found that most countries had widespread availability of at least one first-generation ASM, with carbamazepine being reported as the predominant choice globally. However, second- and third-generation ASMs were found to have limited utilization in low- and middle-income countries [17].

Psychiatric symptoms commonly manifest in individuals with epilepsy, including but not limited to depression, anxiety, and aggression [18]. Children with epilepsy could exhibit symptoms of attention deficit hyperactivity disorder (ADHD) and depressive symptoms [19]. Several studies have investigated the shared genetic and neurobiological mechanisms underlying epilepsy and psychiatric disorders, yet a definitive conclusion has not been reached [20, 21]. An alternative perspective suggests that patients with epilepsy may experience psychiatric side effects after taking ASMs. Several ASMs, including phenobarbital, levetiracetam, gabapentin, felbamate, zonisamide, and topiramate have been associated with cognitive and behavioral adverse effects. Additionally, the use of vigabatrin has been linked to symptoms such as depression, irritability, hyperactivity, increased anxiety levels, psychosis, and insomnia [19]. The implication is that it is crucial to be attentive and vigilant regarding potential psychiatric side effects following the administration of ASMs. The current studies on psychiatric adverse drug reactions (ADRs) caused by ASMs in children are limited, highlighting the need for increased attention and research in this specific area.

The FDA Adverse Event Reporting System (FAERS) is one of the largest pharmacovigilance databases globally, encompassing comprehensive information on all adverse events (AEs) and medication error reports received by the FDA. Therefore, many studies have aimed to assess the potential adverse effects of post-marketing drugs in real-world use using the FAERS database [22, 23].

In this study, we utilized the documented AEs associated with ASMs in children to discern the AEs specifically linked to psychiatric disorders. These investigations may aid in identifying potential psychiatric adverse effects that are not delineated in drug labels, thereby enhancing the management of childhood epilepsy.

## Methods

### Data source

This study downloaded the American Standard Code for Information Interchange (ASCII) report files from the FAERS database for the period from the first quarter of 2004 to the first quarter of 2024. The data were imported into R software version 4.3.0 and processed using the"faersR"package.

We included ASMs that have received FDA approval for the treatment of epilepsy or seizures, as listed in Table S1 [24]. Reports mentioning brand names were reclassified under their corresponding generic names. Duplicate reports were eliminated from the analysis. Only reports suspecting ASMs as the primary drug associated with AEs were considered. Furthermore, this study exclusively focuses on patients below 18 years old. Considering that certain drugs were either not or infrequently used in pediatric patients (< 18 years old), we analyzed a total of 29 ASMs for AEs in the follow-up study.

### Statistical analysis

In this study, the Medical Dictionary for Regulatory Activities (MedDRA) was employed to encode and analyze each preferred term (PT), which was subsequently mapped to the system organ classification (SOC) for further exploration [25]. Firstly, the distribution of SOC features for the 29 ASMs underwent a descriptive analysis. Second, the reporting odds ratio (ROR) [26], proportional reporting ratio (PRR) [27], Bayesian Confidence Propagation Neural Network (BCPNN) [28], and Multi-item Gamma Poisson Shrinker (MGPS) [29] were employed to compute signal values. The utilization of multiple algorithms in combination enables cross-validation to mitigate false positives and enhance the precision of signal detection [30, 31]. The algorithms and criteria are shown in Table 1. The simultaneous fulfillment of four criteria was deemed to be indicative of significant signals.

**Table 1:** Summary of algorithms

Algorithms	Equation^a^	Criteria
ROR	ROR $=\frac{(a/c)}{(b/d)}=\frac{ad}{bc}$	a ≥ 3, 95%CI (lower limit) > 1
	95%CI $={e}^{\text{ln}(ROR)\pm 1.96\sqrt{(\frac{1}{a}+\frac{1}{b}+\frac{1}{c}+\frac{1}{d})}}$	
PRR	PRR $=\frac{a/(a+b)}{c/(c+d)}$	a ≥ 3, 95%CI (lower limit) > 1, PRR ≥ 2, ${\chi }^{2}$ ≥ 4
	95%CI $={e}^{\text{ln}(\text{PRR})\pm 1.96\sqrt{\frac{1}{a}-\frac{1}{a+b}+\frac{1}{c}-\frac{1}{c+d}}}$	
	$${\chi }^{2}=\frac{{\left(ad-bc\right)}^{2}(a+b+c+d)}{(a+b)(a+c)(c+d)(b+d)}$$	
BCPNN	IC $={\text{log}}_{2}\frac{p(x,y)}{p\left(x\right)p(y)}={\text{log}}_{2}\frac{a(a+b+c+d)}{(a+b)(a+c)}$	IC025 > 0
	E(IC)$={\text{log}}_{2}\frac{(a+\gamma 11)(a+b+c+d+\alpha )(a+b+c+d+\beta )}{(a+b+c+d+\gamma )(a+b+\alpha 1)(a+c+\beta 1)}$	
	V(IC)$=\frac{1}{{(\text{ln}2)}^{2}}\left\{\left[\frac{\left(a+b+c+d\right)-a+\gamma -\gamma 11}{(a+\gamma 11)(1+a+b+c+d+\gamma )}\right]+\left[\frac{\left(a+b+c+d\right)-\left(a+b\right)+\alpha -\alpha 1}{(a+b+\alpha 1)(1+a+b+c+d+\alpha )}\right]+\left[\frac{\left(a+b+c+d\right)-\left(a+c\right)+\beta -\beta 1}{(a+c+\beta 1)(1+a+b+c+d+\beta )}\right]\right\}$	
	$$\gamma =\gamma 11\frac{(a+b+c+d+\alpha )(a+b+c+d+\beta )}{(a+b+\alpha 1)(a+c+\beta 1)}$$	
	IC025 $=$ E(IC)−2 $\sqrt{V(IC)}$	
	p.s.$\alpha 1=\beta 1=1; \alpha =\beta =2; \gamma 11=1$	
MGPS	EBGM $= \frac{a(a+b+c+d)}{(a+c)(a+b)}$	EBGM05 > 2
	95%CI $={e}^{\text{ln}(EBGM)\pm 1.96\sqrt{(\frac{1}{a}+\frac{1}{b}+\frac{1}{c}+\frac{1}{d})}}$	

^a^ROR, reporting odds ratio; a, number of reports containing both the suspect drug and the suspect adverse drug reaction; b, number of reports containing the suspect adverse drug reaction with other medications (except the drug of interest); c, number of reports containing the suspect drug with other adverse drug reactions (except the event of interest); d, number of reports containing other medications and other adverse drug reactions; CI, confidence interval; PRR, proportional reporting ratio; χ^2^, chi-square; BCPNN, Bayesian confidence propagation neural network; IC, information component; IC025, the lower confidence interval of IC; MGPS, multi-item gamma Poisson shrinker; EBGM, empirical Bayesian geometric mean; EBGM05, the lower 95% one-sided CI of EBGM

## Results

**Fig. 1:**
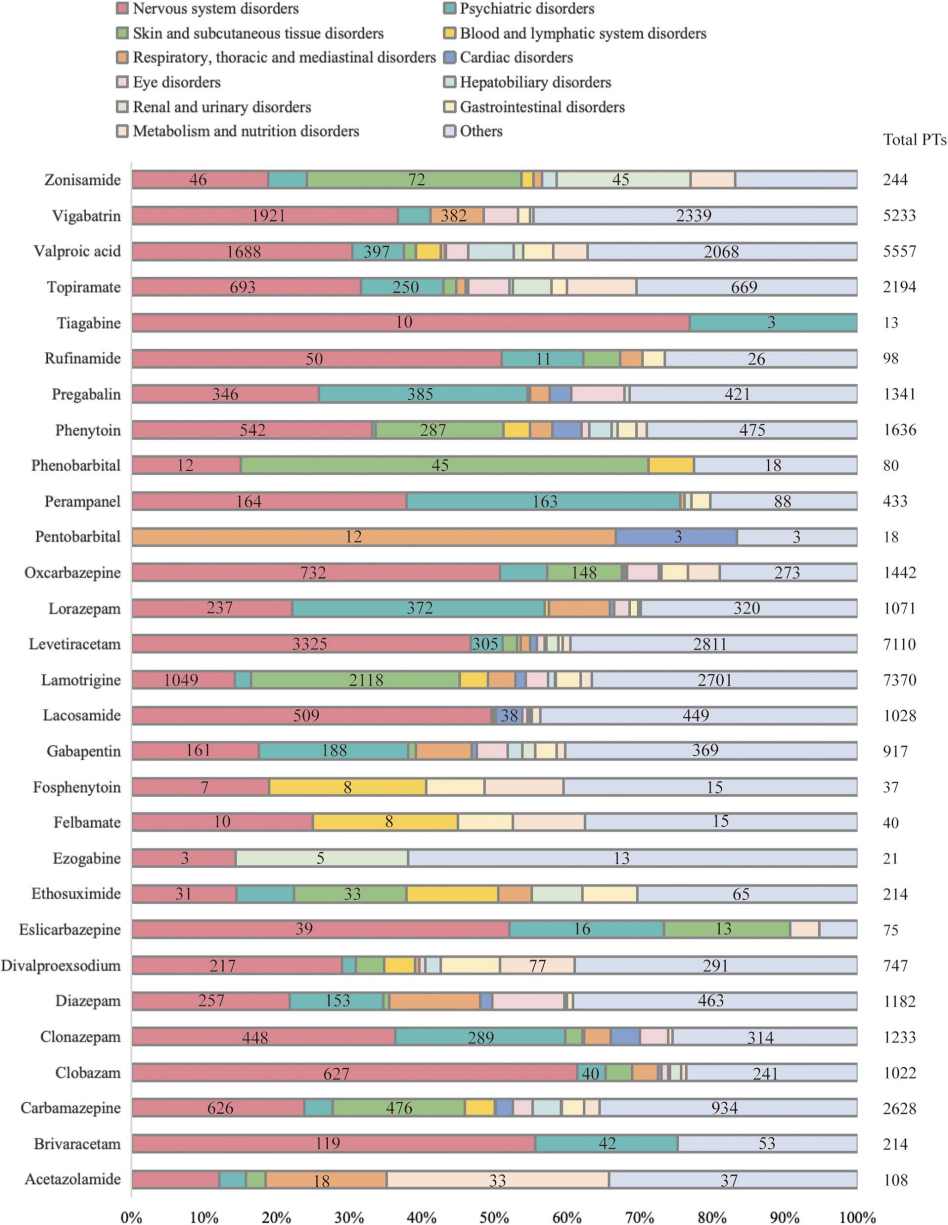
Distribution of AE signals from ASMs in children from the FAERS database, classified based on SOCs level

At the PT level, this study employed four algorithms to analyze ADRs and evaluated compliance with various screening criteria. A total of 2539 PTs were included, involving 25 SOCs (Table S2). We incorporated less involved SOCs. The distribution of SOCs involved in the AEs of 29 ASMs is illustrated in Fig. 1. Nervous system disorders, skin and subcutaneous tissue disorders, and psychiatric disorders are the three most common SOCs when using ASMs in children.

There were 24 ASMs, whose AEs involved psychiatric disorders, totaling 110 PTs and 214 drug-PT relationships (Table S3). The top 30 drug-PT relationships are presented in Table 2, ranked according to the ROR. The drug-PT relationships with the top 5 signal strengths were lorazepam-withdrawal catatonia (*n* = 5, ROR 736.63, PRR 735.15, IC 8.52, EBGM 368.08), levetiracetam-epileptic psychosis (*n* = 3, ROR 270.29, PRR 270.25, IC 6.09, EBGM 68.31), pregabalin-drug use disorder (*n* = 44, ROR 215.33, PRR 211.84, IC 7.33, EBGM 160.87), valproic acid-social (pragmatic) communication disorder (*n* = 4, ROR 169.62, PRR 169.57, IC 6.19, EBGM 73.24), and pregabalin-substance use disorder (*n* = 6, ROR 159.81, PRR 159.46, IC 7.01, EBGM 128.79).

**Table 2:** The top 30 signal strength of psychiatric disorder-related AEs of ASMs in children ranked by ROR at the PTs level in the FAERS database

ASM	PTs	Case Reports	ROR (95% CI)
Lorazepam	withdrawal catatonia	5	736.63 (213.12, 2546.10)
Levetiracetam	epileptic psychosis	3	270.29 (28.11, 2598.63)
Pregabalin	drug use disorder	44	215.33 (153.04, 302.96)
Valproic acid	social (pragmatic) communication disorder	4	169.62 (37.96, 757.95)
Pregabalin	substance use disorder	6	159.81 (65.51, 389.88)
Levetiracetam	automatism epileptic	8	120.16 (41.69, 346.34)
Topiramate	self-induced vomiting	6	107.56 (41.72, 277.30)
Lorazepam	alcohol abuse	5	96.92 (38.12, 246.45)
Levetiracetam	childhood psychosis	3	90.10 (18.18, 446.42)
Gabapentin	psychiatric decompensation	3	79.39 (23.82, 264.56)
Clonazepam	substance use disorder	3	67.69 (20.57, 222.79)
Topiramate	alice in wonderland syndrome	6	62.05 (25.53, 150.81)
Lamotrigine	poverty of speech	8	60.25 (24.23, 149.8)
Lorazepam	disorganised speech	5	57.55 (23.14, 143.11)
Pregabalin	drug abuse	208	56.71 (48.98, 65.66)
Clonazepam	drug use disorder	14	52.85 (30.6, 91.27)
Valproic acid	executive dysfunction	3	47.70 (12.65, 179.83)
Gabapentin	personality disorder	10	46.22 (24.28, 87.98)
Lorazepam	generalised anxiety disorder	4	43.96 (16.02, 120.64)
Valproic acid	impaired reasoning	6	38.17(15.33, 95.06)
Carbamazepine	poverty of speech	3	37.34 (10.88, 128.18)
Valproic acid	learning disorder	51	37.19 (27.22, 50.83)
Valproic acid	automatism	5	35.34 (13.12, 95.20)
Lamotrigine	asocial behavior	9	33.89 (15.60, 73.610)
Clonazepam	mutism	6	33.88 (14.89, 77.09)
Diazepam	fear of death	3	33.82 (10.62, 107.72)
Lamotrigine	factitious disorder	3	31.06 (8.24, 117.08)
Perampanel	homicidal ideation	4	30.88 (11.46, 83.24)
Topiramate	bradyphrenia	18	30.30 (18.60, 49.36)
Lorazepam	persecutory delusion	3	29.44 (9.28, 93.41)

Based on the Diagnostic and Statistical Manual of Mental Disorders-5 th Edition (DSM-5) and International Classification of Diseases-10 th Edition (ICD-10), 110 PTs related to psychiatric disorders were classified into 10 categories for facilitating analysis and comprehension, which encompassed behavioral and impulse control disorders, cognitive and learning disorders, language and speech disorders, autism spectrum disorder related symptoms, mood and emotional disorders, psychotic symptoms, sleep disorders, substance dependence and abuse, suicidal and self-injurious behavior, and other neuropsychiatric symptoms (Table S4-5).

**Fig. 2:**
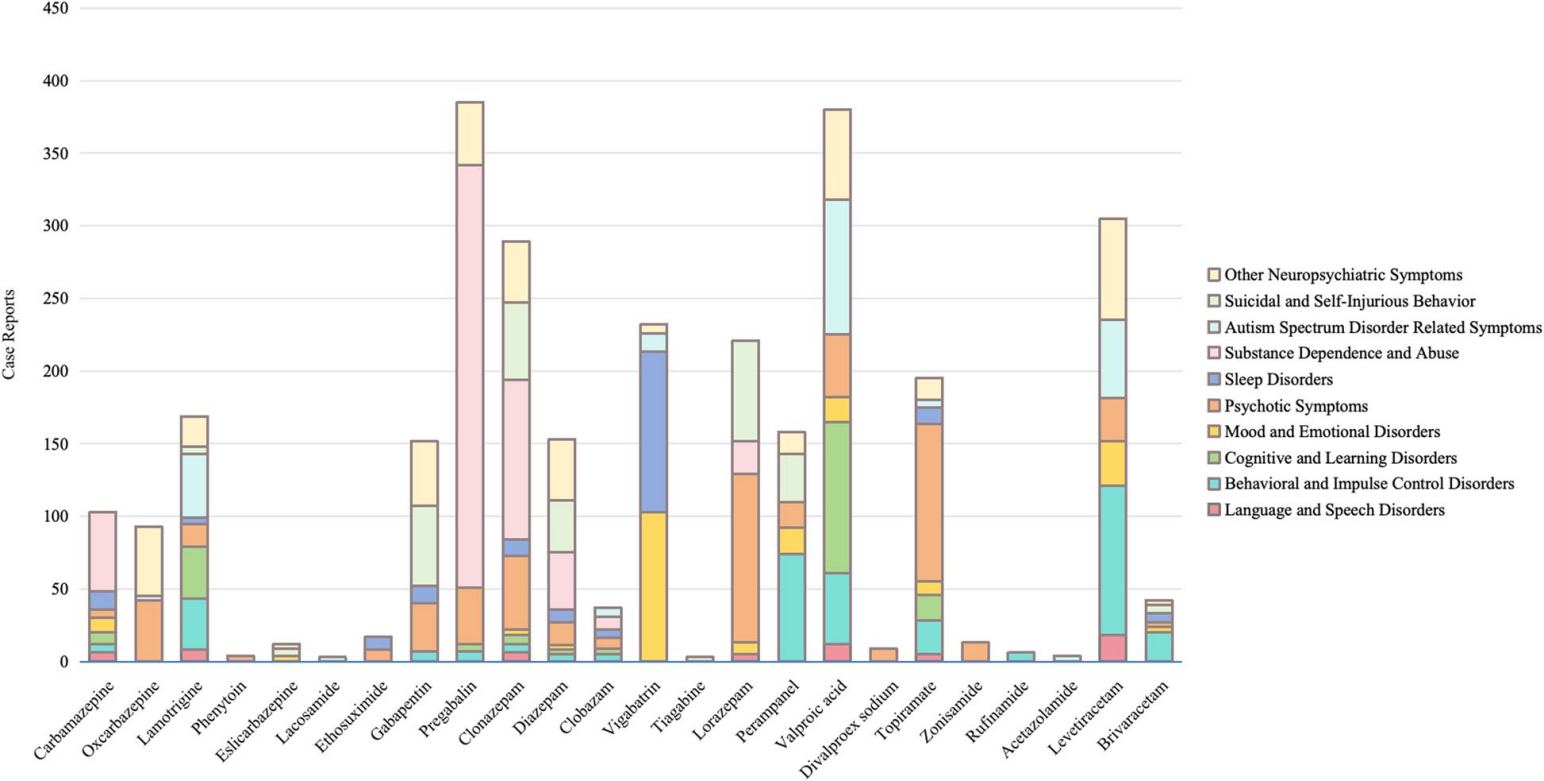
Distribution of psychiatric disorder-related AEs of ASMs in children from the FAERS database

Psychotic symptoms (mainly in lorazepam and topiramate, *n* = 116 and *n* = 109), substance dependence and abuse (mainly in pregabalin and clonazepam, *n* = 291 and *n* = 110), and the other neuropsychiatric symptoms (mainly seen in levetiracetam and valproic acid, *n* = 70 and *n* = 62) were the common types of psychiatric disorder-related AEs of ASMs in children. Behavioral and impulse control disorders were reported more frequently in levetiracetam and perampanel (*n* = 103 and *n* = 74). Cognitive and learning disorders were mainly found in valproic acid and lamotrigine (*n* = 104 and 36). Vigabatrin reported more mood and emotional disorders (*n* = 103) and sleep disorders (*n* = 110). Autism spectrum disorder related symptoms are mainly seen in valproic acid, levetiracetam, and lamotrigine (*n* = 93, *n* = 54, and *n* = 41) (Fig. 2).

**Fig. 3:**
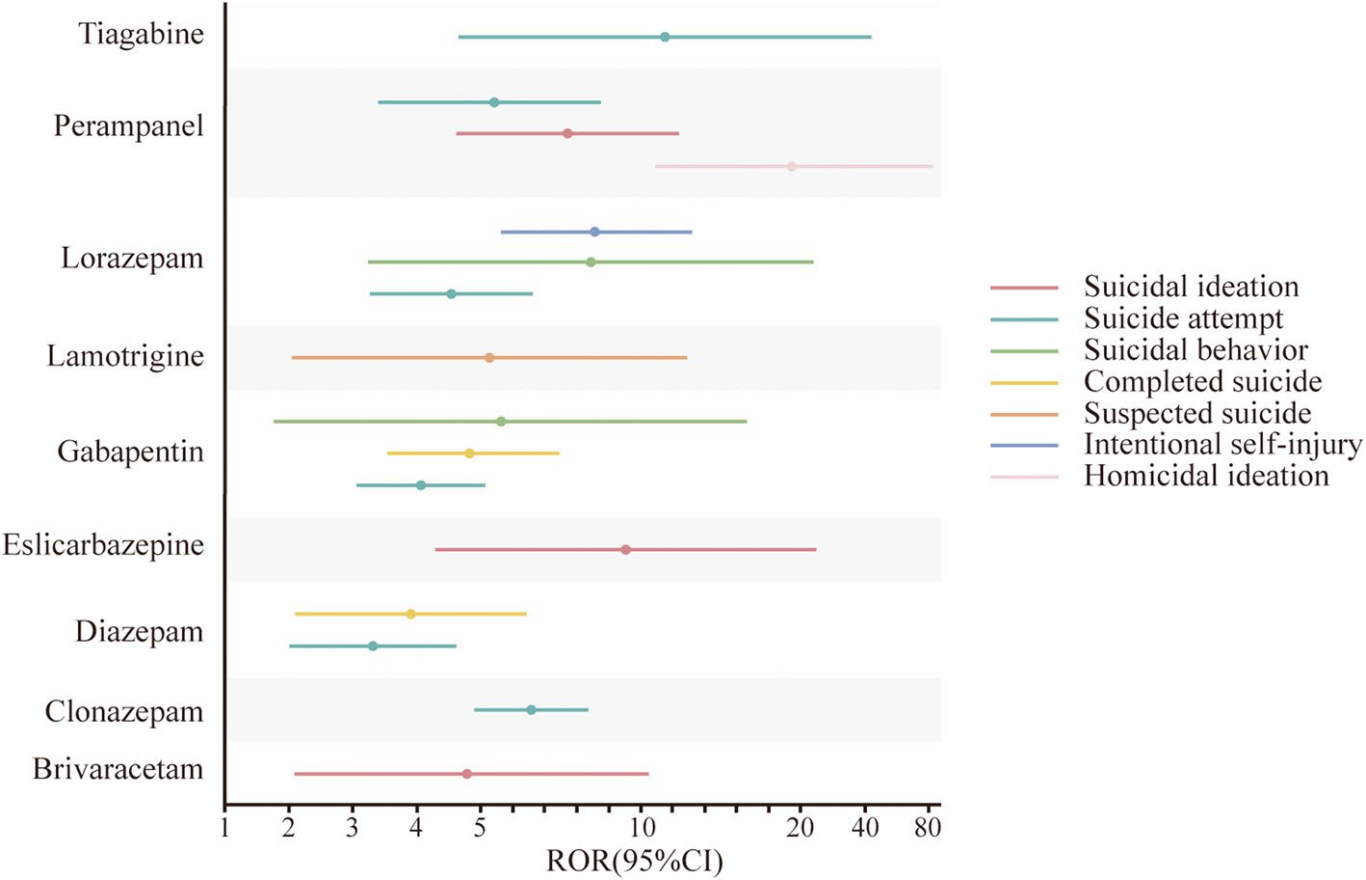
The associations between ASMs and AEs of suicidal and self-injurious behavior, based on ROR

Surprisingly, nine ASMs (brivaracetam, clonazepam, diazepam, eslicarbazepine, gabapentin, lamotrigine, lorazepam, perampanel, and tiagabine) were associated with suicidal and self-injurious behavior in children. The drug-PT relationship with the highest signal strength was perampanel-homicidal ideation (*n* = 4, ROR 30.88, PRR 30.75, IC 4.92, EBGM 30.19). The most frequent was clonazepam-suicide attempt (*n* = 53, ROR 6.30, PRR 6.21, IC 2.62, EBGM 6.16) (Fig. 3 and Table S5).

## Discussion

Children diagnosed with epilepsy frequently exhibit comorbid neuropsychiatric developmental disorders, including cognitive impairments, behavioral dysfunctions, psychiatric manifestations (such as anxiety and depression), language deficits, and sleep disorders. These associations may be attributed to specific childhood-onset epilepsy syndromes characterized by severe epileptic seizures and concurrent neurodevelopmental disorders, such as Dravet syndrome (DS) or Lennox-Gastaut syndrome (LGS) [32]. The frequent seizures may also contribute to the development of psychiatric disorders. The nocturnal seizures can intensify sleep disorders, exacerbating cognitive and behavioral impairments [33]. Apart from disease mechanisms, ASMs are also linked to a range of behavioral and psychological AEs, including irritability, attack, mood disorders, depression, and anxiety [32]. In this study, we analyzed psychiatric disorders related to AEs of ASMs in children using reports collected from the FAERS database, aiming to provide valuable insights for clinical decision-making.

The FAERS database remains a fundamental component of post-marketing pharmacovigilance, widely used to monitor drug safety and facilitate timely identification of potential safety concerns. It has been extensively utilized for evaluating the real-world potential adverse effects of ASMs [24, 34]. We used four methods to mine potential AE signals to increase the stability of the results. In the case of the most commonly used ROR, a high ROR value typically indicated a robust association between the drug and a specific AE. This association was further substantiated when accompanied by a substantial number of case reports (such as pregabalin-drug use disorder, *n* = 44, ROR 215.33). High ROR values combined with a low number of case reports suggest a potential association, but additional data are required for confirmation (such as lorazepam-withdrawal catatonia, *n* = 5, ROR 736.63). A low ROR value coupled with a high number of cases has been reported, which may indicate that the AEs represent various common drug reactions; however, caution should still be exercised due to the reporting numbers (such as vigabatrin-irritability, *n* = 103, ROR 4.47). Therefore, the AE signals that meet the screening criteria should be considered significant in clinical practice.

A study involving 4085 adult patients newly initiated with ASM for epilepsy revealed that 17.2% of the patients experienced psychiatric and behavioral adverse effects, which were deemed intolerable by 13.8% of them. The study revealed that patients taking levetiracetam and zonisamide were more susceptible to experiencing adverse psychiatric and behavioral reactions. Levetiracetam primarily exhibited symptoms of irritability, aggression, other behavioral issues, depressive mood, and anxiety. Zonisamide mainly manifested as depressive mood and psychosis [35]. Our study also found that levetiracetam had the highest case reports related to behavioral and impulse control disorders, primarily encompassing behavior disorders, impulse-control disorders, and hostility. Zonisamide also included AEs associated with psychotic symptoms (hallucination, visual and confusional state). Levetiracetam, perampanel, brivaracetam, valproic acid, and clonazepam were found to be associated with psychobehavioral AEs, such as aggression and irritability [32]. Previous studies have suggested that lamotrigine can improve aggressive behavior in epilepsy patients [36]. On the other hand, it has also been found that lamotrigine may exacerbate aggression and violent behavior in some patients [37]. In this study, lamotrigine was found to be associated with antisocial behavior and conduct disorders, which were less frequently observed in previous research.

Valproic acid, lamotrigine, and topiramate reported the highest number of cognitive and learning disorder AEs in our study. Valproic acid was considered to be associated with an increased risk of autism spectrum disorder, but there were few studies on its effect on cognitive function [32]. It is important to note that the observed association may also be influenced by prescribing bias. Valproic acid is commonly used in clinical practice for children with epilepsy and co-occurring autism spectrum (ASD) disorder due to its mood-stabilizing properties, which may contribute to improvements in core ASD symptoms [38]. Several studies have reported a partial improvement in cognitive function among patients following lamotrigine therapy [39, 40]. A study of 87 children treated with topiramate reported that the main reason for discontinuation was deemed to be unacceptable cognitive dulling (27, 31%) [41]. The variations in cognitive and learning outcomes may be associated with the stage of disease progression and the demographic characteristics of the population.

Pregabalin, lorazepam, clonazepam, diazepam, phenytoin, clobazam, and carbamazepine were reported with substance dependence and abuse. Pregabalin has been suggested to have a modulatory effect on GABA and glutamate systems, leaving room for drug dependence [42]. In one study, the likelihood of remaining drug-free dependent for 5 years of treatment with clobazam and clonazepam was 99.4% and 98.5%, respectively, in patients with epilepsy [43]. The prescription should still be monitored for signs of abuse, particularly in patients with a prior history of substance misuse.

It is worth noting that vigabatrin reported irritability (*n* = 103, ROR 4.47) and insomnia (*n* = 99, ROR 3.83) of the AEs. In a study of 135 children treated with vigabatrin as adjunctive therapy, agitation and insomnia were reported in 8.8% of patients, while somnolence was observed in 6% [44]. In addition, among the AEs regarding vigabatrin, death was reported (*n* = 447, ROR 14.11) (Table S2). Although this is not the primary focus of this research, it is worth conducting a follow-up study to further explore and provide cautionary insights.

Perampanel, tiagabine, eslicarbazepine, lorazepam, clonazepam, gabapentin, lamotrigine, brivaracetam, and diazepam were associated with suicidal and self-injurious behavior. In a case–control study, the utilization of ASMs was found to be associated with an elevated risk of suicide (mortality rate ratio, MRR = 1.26, 95%CI = 1.13–1.40) [45]. The results of a meta-analysis revealed no evidence suggesting that ASMs (including eslicarbazepine, perampanel, and brivaracetam) were associated with an increased risk of suicidal ideation or attempts [46]. Despite the absence of convincing evidence linking ASMs to an elevated risk of suicidal and self-injurious behavior, it is still imperative to remain vigilant regarding these potential AEs.

We also identified some rare AEs that deserve attention and vigilance. For example, lamotrigine-related Munchausen's syndrome (*n* = 4, ROR 15.00), a mental disorder in which one fantasizes about being sick to seek medical attention or be hospitalized; valproic acid-related automatism (*n* = 5, ROR 35.34), the unconscious or uncontrolled state in which certain movements or behaviors are performed. These AE signals played a crucial role in guiding the assessment of drug safety, risk management, and clinical decision-making.

Caution is warranted when interpreting the associations between certain ASMs and psychiatric AEs, particularly for agents like lamotrigine and valproic acid, which are also recognized for their mood-stabilizing properties. Lamotrigine, beyond its broad-spectrum efficacy in seizure control, is approved by the FDA for the treatment of bipolar disorder and has shown promise in managing affective symptoms in pediatric epilepsy populations [47, 48]. Valproic acid, similarly, is frequently prescribed in children with epilepsy and co-occurring ASD, owing to its dual role in seizure suppression and positive modulation of behavioral symptoms, which may contribute to improvements in core ASD features [38]. Therefore, the observed associations—such as lamotrigine with suicidality and valproic acid with ASD—may not reflect true adverse drug effects but rather result from prescribing bias, wherein these medications are preferentially selected for patients with specific neuropsychiatric comorbidities. These considerations underscore the importance of interpreting such associations with care and highlight the need for future studies that integrate detailed clinical and diagnostic information to distinguish causal drug effects from treatment indications.

ASMs exert their antiepileptic effects through multiple mechanisms and by targeting a range of molecular substrates, including the enhancement of GABAergic inhibition, suppression of glutamatergic excitation, direct modulation of synaptic release, and regulation of voltage-gated ion channel conductance [49]. Notably, these biological targets are also critically involved in the regulation of mood and behavior [50], which may partly explain why ASMs are associated with specific potential neuropsychiatric ADRAs. Studies have shown that some ASMs enhancing GABAergic transmission, such as phenobarbital, benzodiazepines, topiramate, and tiagabine, may induce GABAergic hyperactivity, disrupting mood regulation and leading to neuropsychiatric symptoms like depression, irritability, impulsive aggression, and hyperactivity [51, 52]. These effects are more common in children and individuals with a prior history of psychiatric disorders [53], highlighting the role of individual susceptibility in ASM-related neuropsychiatric reactions. Alterations in the 5-HT (serotonin) and glutamatergic pathways have also been implicated in ASM-related neuropsychiatric effects, particularly in the manifestation of aggressive behavior, including agitation, anger, hostility, and overt aggression, which may, in some cases, contribute to an increased risk of suicidality [54, 55]. In some patients, the rapid suppression of seizures can trigger psychiatric symptoms, a phenomenon known as "forced normalization" [56]. This paradoxical reaction, often associated with psychosis, hypomania, or depression, is thought to involve disrupted balance between excitatory and inhibitory activity in limbic circuits. Forced normalization has been reported with many ASMs and may, at least in part, explain their paradoxical exacerbation of psychiatric symptoms in patients with epilepsy [50]. The underlying pathophysiological mechanisms, however, have not been fully characterized.

This study employed real-world data from the FAERS database to investigate AEs related to psychiatric disorders in children associated with ASMs, providing valuable insights and guidance for clinical decision-making. Given the identified associations between ASMs and neuropsychiatric AEs, it is crucial for clinicians to remain vigilant when prescribing these medications, especially in pediatric populations. Healthcare providers should closely monitor children on ASMs for any early signs of mood disturbances, irritability, aggression, or other behavioral symptoms, particularly in those with a history of psychiatric disorders. Regular follow-up and timely intervention are essential to mitigate these adverse effects.

However, several limitations should be acknowledged. Firstly, the FAERS database functions as a spontaneous submission system, rather than imposing strict censorship on all submitted reports, which may lead to reporting bias and information gaps. Despite the standardization of MedDRA terms, variations in symptom descriptions and missing data (such as age or medication doses) may exist. Additionally, reporting bias, such as the overreporting of severe AEs may occur. The absence of denominator data—i.e., the total number of individuals exposed to a specific drug—prevents the estimation of incidence rates or risk quantification. Furthermore, essential covariates such as polypharmacy, age, and gender were often unavailable or incompletely reported, precluding meaningful adjustment for these potential confounders. As a result, while the detected AE signal can only suggest a statistical association between the drug and the AE, indicating a potential cause, it does not support causal inference. These limitations highlight the importance of interpreting findings alongside complementary clinical, epidemiological, and pharmacological evidence. Second, the use of strict statistical thresholds in signal detection may lead to false-negative results, particularly for rare or underreported events. Such vulnerability is inherent to spontaneous reporting systems and warrants further exploration through sensitivity analyses or complementary approaches such as Natural Language Processing (NLP) of narrative fields. Third, the FAERS report primarily originates from the United States and Europe. The findings may not be generalizable due to ethnic and regional variability in drug response. To improve robustness, future studies should incorporate multiple pharmacovigilance systems (e.g., EudraVigilance, WHO VigiBase) and validate signals using structured clinical data such as electronic health records. Fourth, this study focused on the utilization of a single ASM for signal detection and screening, without considering the potential impact of drug interactions on ADRs. Fifth, while disproportionality analysis remains a powerful tool for detecting ADR signals [57]—particularly rare or unexpected events—it should be noted that it does not constitute a definitive measure of causality or risk. Currently, there is no universally accepted gold standard for ADR signal detection. These methods primarily serve as indicators of potential safety concerns, and their interpretation must be supported by clinical data, case-level assessment, and complementary research approaches [58]. Future studies should focus on establishing standardized methodologies for signal detection to ensure consistency and accuracy across pharmacovigilance research. It is worth mentioning that AEs during antiepileptic therapy may reflect genetic susceptibility. Pathogenic variants in neurodevelopmental genes can simultaneously predispose to epilepsy and comorbid neuropsychiatric traits, such as intellectual disability or autism spectrum features, through shared biological pathways [59]. This highlights the need for integrating genetic profiling into pharmacovigilance studies of drug-related neuropsychiatric manifestations. Future research should focus on identifying rare genetic variants and susceptibility loci to elucidate molecular mechanisms underlying these adverse drug responses.

## Conclusions

In this study, the real-world safety characteristics of ASMs were assessed in terms of pharmacovigilance signals by conducting signal detection analysis using the FAERS database. Our focus was on AEs related to psychiatric disorders caused by ASMs in pediatric patients. Although these findings were limited to statistical correlations, they provided valuable information for clinical medication practices aimed at enhancing rationalization and safety measures. Close monitoring is recommended for children receiving ASMs, and if any significant ADRs occur, healthcare providers should be involved to consider dose adjustments or discontinuation of treatment.

## Supplementary Information

Supplementary Material 1.

## Data Availability

The public can obtain all reported AEs by accessing the official FDA website (https://fis.fda.gov/extensions/FPD-QDE-FAERS/FPD-QDE-FAERS.html). The dataset generated during and analyzed during the current study are available from the corresponding author on reasonable request.

## Abbreviations

ADHD: Attention deficit hyperactivity disorder
ADRs: Adverse drug reactions
AEs: Adverse events
ASCII: American Standard Code for Information Interchange
ASD: Autism spectrum disorder
ASMs: Antiseizure medications
BCPNN: Bayesian confidence propagation neural network
DALYs: Disability-adjusted life years
DS: Dravet syndrome
DSM-5: Diagnostic and Statistical Manual of Mental Disorders-5th Edition
FAERS: FDA Adverse Event Reporting System
ICD-10: International Classification of Diseases-10th Edition
ILAE: International League Against Epilepsy
LGS: Lennox-Gastaut syndrome
MedDRA: Medical Dictionary for Regulatory Activities
MGPS: Multi-item gamma Poisson shrinker
NLP: Natural language processing
PRR: Proportional reporting ratio
PT: Preferred term
ROR: Reporting odds ratios
SOC: System organ classification
